# Quality of Life and Mental Distress in Patients with Chronic Low Back Pain: A Cross-Sectional Study

**DOI:** 10.3390/ijerph191710657

**Published:** 2022-08-26

**Authors:** Dijana Hnatešen, Roman Pavić, Ivan Radoš, Iva Dimitrijević, Dino Budrovac, Maja Čebohin, Ivana Gusar

**Affiliations:** 1 Faculty of Medicine, Josip Juraj Strossmayer University of Osijek, 31000 Osijek, Croatia; 2 Nursing Institute “Professor Radivoje Radić”, Faculty of Dental Medicine and Health Osijek, Josip Juraj Strossmayer University of Osijek, 31000 Osijek, Croatia; 3 Clinical Department of Pain Management, University Hospital Osijek, 31000 Osijek, Croatia; 4 Clinical Hospital of Traumatology, University Hospital Centre “Sestre Milosrdnice”, 10000 Zagreb, Croatia; 5 Medical School Osijek, 31000 Osijek, Croatia; 6 Department of Health Studies, University of Zadar, 23000 Zadar, Croatia

**Keywords:** chronic pain, low back pain, quality of life, SF-36, pain measurement, CORE-OM

## Abstract

The aim of this study was to examine the levels of health-related quality of life (HRQoL), pain intensity, and mental distress in participants with chronic low back pain (CLBP), and to examine the differences in the HRQoL of participants with respect to mental distress and the correlations of the examined variables. Data were collected from 148 patients using the SF-36 Health Status Questionnaire (SF-36), the Clinical Outcomes in Routine Evaluation–Outcome Measure (CORE-OM) questionnaire, and the visual-analog pain scale (VAS). The results indicate poorer self-assessment of physical health (M_e_ = 28.1) compared to mental health (M_e_ = 39.4). Participants with higher levels of mental distress reported significant emotional limitations (*p* = 0.003), lower energy (*p* < 0.001), poorer psychological health (*p* < 0.001) and social functioning (*p* < 0.001), more pain (*p* = 0.007), and, ultimately, poorer general health (*p* < 0.001). The level of mental distress was related to the level of HRQoL, while a correlation with the level of pain of the participants was not found. The study results indicate a connection between the presence of mental distress and almost all aspects of HRQoL in participants with CLBP.

## 1. Introduction

Chronic pain (CP) is a global problem that can permeate all aspects of life [1,2,3]. It affects the whole person, his/her physical health, psychological well-being, and psychosocial problems, and it brings with it a future filled with depression, melancholy, hopelessness, loneliness, a loss of identity, and a low quality of life [3]. Globally, it is estimated that one in five adults in Europe suffer from chronic or recurrent pain [4,5], and that each year, one in ten adults worldwide are diagnosed with chronic pain [6]. For more than twenty years, the International Association for the Study of Pain (IASP) defined CP as pain that persists beyond normal tissue healing time, which is assumed to be three months [7] and as an aversive sensory and emotional experience that is typically caused by, or resembling that caused by, actual or potential tissue injury [8]. Until May 2019, diagnoses of CP were not systematically represented in the International Classification of Diseases (ICD-10) [8]. This has changed with the adoption of the ICD-11 by the World Health Organization (WHO), and the ICD-11 was the first version to include CP [8]. Therefore, CP is now regarded as not so much a symptom of disease, but as a disease in itself. Classifications of CP are based on current scientific evidence and a biopsychosocial model [8]. CP can cause maladaptive cognition and behaviors that, in turn, can worsen daily function, increase psychological stress, and even prolong the pain itself [9]. Fatigue, irritability, sleep, and decreased appetite disorders are somatic symptoms that often accompany CP. In CP, emotional, motivational, cognitive, and psychosocial factors may be more intense than nociceptive pain alone [10]. Health-related quality of life (HRQoL) and mental distress are two central areas that are constantly recurring in some form; they reflect perceived functioning and well-being in the physical, mental, and social dimensions of health and feelings of depression and anxiety. HRQoL and mental distress are recommended as core areas of the outcomes in clinical trials of pain management interventions to increase research consistency [11,12,13]. The most commonly used questionnaire as an indicator of HRQoL is the Short Form-36 Health Status Questionnaire (SF-36) [14,15]. The multidimensional negative impacts of CP lead to poorer HRQoL among patients with CP compared to the general population and patients with other chronic diseases [16]. For a better understanding, it is necessary to define certain terminology. According to IASP, back pain consists of pain in the cervical, thoracic, lumbar, and/or sacral regions [17]. Low back pain (LBP) is anatomically defined as extending from the 12th rib to the iliac crest [18], comprising soft tissue, vertebrae, zygapophyseal and sacroiliac joints, intervertebral discs, and neurovascular structures, and each of these, alone or in combination, can contribute to LBP [19] which, in order to be chronic, must, according to the IASP, last longer than three months [8]. Research has shown that although many episodes of LBP improve substantially within six weeks and 33% of patients recover in the first three months, 65% still report some pain at 12 months [20,21,22]. Furthermore, up to 33% of people will have a recurrence within one year of recovering from a previous episode [20,23]. The prevalence of chronic back pain increases linearly from the third decade of life to age 60, with a higher prevalence in females [24]. Among all CP problems and spinal pain conditions, LBP is the most common and important clinical, social, economic, and public health problem, affecting the population indiscriminately across the world [25]. LBP occurs in about 60–80% of people at some point in their lives, and it can begin in childhood [26,27]. The prevalence of LBP that limits activity was estimated at 7.3% globally in 2015, meaning that at that time, approximately 540 million people worldwide were affected by LBP [20], while the estimates of lifetime prevalence range from 39% to 83% [28]. For nearly all people with LBP, it is not possible to identify a specific nociceptive cause. Only a small proportion of people have a well-understood pathological cause [20]. The proportion of people presenting to primary care with a specific identifiable cause of LBP is estimated to be 0.7–4.5% with osteoporotic vertebral fractures, 5% with inflammatory spondyloarthropathies, 0.0–0.7% with malignancy, and 0.01% with infections [29]. In addition, Russo et al., state that LBP is primarily caused by intervertebral disc degeneration [30]. Some other causes of LBP can be metabolic bone disease, failed spinal surgery, congenital and acquired disc disease, and lumbar muscle spasm [7]. CLBP is one of the most prevalent CP disorders associated with a high burden on individuals and society, and it can have a significant influence on an individual’s HRQoL [31], such as a high intensity of pain and disability, a lower prognosis rate, significant physical limitations [32], and an inability to work [33].

Data obtained from the Global Burden of Disease Study 2016 identified that the leading cause of disability and the disease burden worldwide is precisely the high prevalence of pain and pain-related illnesses [34]. Over 80% of the total costs attributable to LBP are due to indirect costs such as the loss of productivity and disability payments in countries that have functioning social welfare systems [35,36]. Further, it is known that psychological factors are an important domain in CLBP to assess treatment effectiveness [37]. Hong et al., studied depression, anxiety, disability, and HRQoL in patients with CLBP and found these patients to have considerable functional disability and significant impairment of psychological status, with a low HRQoL [38]. Marčić et al., studied the prevalence of depression and the relationship between depression and pain intensity in 99 LBP patients. While their general physical symptoms were mostly common (71%), these were closely followed by anxiety (70%) and depressed mood (67%). They concluded that depression was more severe in LBP patients with severe disease compared with those with mild or moderate disease [39]. Access to effective pain management techniques may be considered to be a fundamental human right [40], but up to 68% of CP sufferers describe their pain as not adequately controlled [4]. The adequate management of CP is not only a moral and ethical imperative, it also mitigates a sufferer’s subsequent physical and psychological complications [41,42].

Considering the lack of similar studies in the Republic of Croatia, as well as the frequency, importance, and impact of CLBP on the HRQoL of ill people, the aim of this study was to examine the level of HRQoL associated with the intensity of pain and the level of mental distress in participants with CLBP and to examine the differences in the HRQoL with respect to mental distress and the correlation between the examined variables in participants with CLBP.

## 2. Materials and Methods

This cross-sectional study was conducted between December 2020 and January 2022 during participant examinations at the Clinical Department of Pain Management at the University Hospital Osijek. The study was conducted with the approval of the Ethics Committee (R1: 13800-3/2020) and in accordance with the Declaration of Helsinki. The criteria according to which participants were chosen were that participants must be 18 years of age and older, have chronic LBP (pain ≥ 3 months), do not suffer from cognitive and/or mental disease, and are able to communicate in Croatian. Out of 217 participants, 175 met the inclusion criteria and, after being informed about the aims of the study, voluntarily agreed to participate in the study. The participants were also asked for written consent to participate in the study. The questionnaires were given to the participants by the authors, and after filling them out, the participants brought back the questionnaires to the Clinical Department of Pain Management at the University Hospital Osijek. Out of the 175 participants who were given the questionnaires, 157 returned filled-out questionnaires. Nine of them were excluded for having partially solved questionnaires.

### 2.1. SF-36 Questionnaire

The Croatian version of the SF-36 Health Status Questionnaire was used to measure HRQoL [43,44]. The questionnaire was used for the self-assessment of mental and physical health and social functioning. It consists of 36 items, and each of the items refers to one of eight different areas of health within two major concepts (mental and physical health). The questionnaire contains different health scales: 1. physical functioning (consists of 10 items), 2. physical limitations (4 items), 3. emotional limitations (3 items), 4. social functioning (2 items), 5. mental health (5 items), 6. energy (4 items), 7. pain (2 items), and 8. general health (5 items). The result, on an individual scale, was expressed as a standardized value for each dimension, ranging from 0 to 100. The internal consistency of the SF-36 scales ranged from 0.78 to 0.94. The higher a result is on an individual scale, the better the person has assessed this dimension [43,44].

### 2.2. CORE-OM Questionnaire

The Croatian version of the CORE-OM (Clinical Outcome in Routine Evaluation—Outcome Measures) questionnaire was used to measure general mental distress [45]. The questionnaire contains 34 statements, and for each of them, the respondents estimate how often they felt the way described during the last week on a scale from 0 to 4 (from 0—never to 4—almost always). The questionnaire measures four dimensions: 1. well-being (4 statements, e.g., I was satisfied with myself), 2. problems/symptoms (12 statements, e.g., I was tormented by pain or other physical problems), 3. functioning (12 statements, e.g., I was able to do almost everything I needed to), and 4. risky behaviors (6 claims, e.g., it occurred to me to get hurt). The total result is used most often, which is obtained by summing the answers on all items. A higher overall score indicates that the person is more anxious and has more problems. The results can also be presented as the total average result, which is obtained by dividing the total result by the number of items. The questionnaire’s satisfactory internal consistency and test–retest reliability, as well as good convergent validity, were confirmed. The scores were the same for men and women, and on this basis, it is possible to identify people with severe mental disorders. The borderline value was 1.38, with higher scores indicating more distress [45].

### 2.3. VAS

The visual-analog scale of pain (VAS) was used to measure the intensity of pain. The test consists of a solid line bounded at the beginning and end of its length by numbers from 0 to 10. On the far left is the number 0, which indicates the absence of pain, while on the far right is the number 10, which indicates unbearable pain. The VAS is probably the most commonly used measure of pain in clinical practice, with a high degree of resolution [46]. The task of the participants is to mark the intensity of their pain on the scale by rounding off the number.

### 2.4. Statistical Methods

Standard statistical methods were used for statistical analysis. All collected categorical data are presented with absolute and relative frequencies. The normality of the distributions was tested using the Kolmogorov–Smirnov test (K-S). Numerical data were presented with median and interquartile ranges, as the distributions within the parameters did not follow normal Gaussian distributions. For testing statistically significant differences between groups of participants, the Mann–Whitney U test was used, while for correlation analysis, Spearman’s coefficient of rank correlation test was used. Statistical analysis was performed with the IBM SPSS Statistics (release 24.0.0.0; IBM Corp., 2016. IBM SPSS Statistics for Windows, IBM Corp., Armonk, NY, USA) software tools, with statistical significance defined as α < 0.05.

## 3. Results

This study involved 148 participants. The median age was 57.5 years (interquartile range from 49 to 65 years) in a range of 28 to 79 years. Furthermore, we recorded socio-demographic characteristics such as gender, age, education, employment, and marital status (Table 1).

The distribution of scores for each scale of the SF-36 Health Status Questionnaire showed the highest median score for psychological health (56.0) and the lowest median score (0.0) for both physical limitations and emotional limitations (Table 2). The median score for the CORE-OM total was 1.24, which is below the borderline value of 1.38, while the median score for the well-being (1.75) and problems (1.79) subscales were above the borderline value. (Table 2).

A statistically significant difference in the SF-36 Health Status Questionnaire subscales considering a high and a low level of mental distress (CORE-OM) was found between the groups of participants. The participants who had high levels of mental distress noted significant emotional limitations (*p* = 0.003), lower energy (*p* < 0.001), poorer psychological health (*p* < 0.001) and social functioning (*p* < 0.001), stronger pain (*p* = 0.007), and poorer general health (*p* < 0.001) in comparison with participants who had low levels of mental distress (Table 3).

A moderate negative correlation was found between the well-being dimension of the CORE-OM and all subscales of the SF-36 Health Status Questionnaire, except for physical functioning and limitation. A moderate negative correlation was found between the CORE-OM problems and functioning dimensions and all of the subscales of the SF-36 Health Status Questionnaire, except for physical limitation and functioning. The CORE-OM total had a significant weak negative correlation with the SF-36 Health Status Questionnaire subscales of emotional limitations, energy, psychological health, social functioning, pain, and general health. Moreover, a weak negative correlation was found between the VAS and SF-36 physical functioning (*r* = −0.28), between the VAS and SF-36 physical limitations (*r* = −0.19), and between the VAS average and the SF-36 Health Status Questionnaire pain subscale (*r* = −0.34) (Table 4).

## 4. Discussion

The aim of this study was to examine the level of HRQoL, intensity of pain, and level of mental distress in participants with CLBP and to examine the differences in the HRQoL of participants with respect to mental distress and the correlation of the examined variables. The sample included in this study involved participants who were similar in the average age of the general adult population in the Republic of Croatia [47]. The largest share of the participants had completed secondary education and were retired, which also corresponded to the distribution of the adult population in the Republic of Croatia [47]. Similar participant characteristics were described in other studies that indicated significant differences between genders regarding prevalence, degree of disability, and number of comorbidities, which are all higher in individuals who identify as women [48]. Our sample was dominated by women, too.

The results obtained in this research confirm the presence of moderate pain and a poorer self-assessment of physical health in comparison to mental health in patients with CLBP, which is in accordance with other studies [49,50]. Namely, our participants rated the worst in the area of physical functioning, i.e., they stated that the difficulties present in their physical functioning leads them to shorten the time they spend working or cause them difficulties such that they cannot perform their planned activities. Similar results were published in a study of 30,074 workers that examined the risk of certain occupations for the occurrence of LBP [51]. Martin et al., also reported a significant presence of physical limitations in patients with spine problems [52]. In the research conducted by Martinec et al., it was determined that patients with rheumatoid arthritis have the worst results in the area of physical limitations and physical functioning, while they achieve the best results in the area of psychological health [53], which partially agrees with our results in relation to the subscales that have the worst impact on HRQol in contrast to psychological health, which both groups estimate to have the least impact on HRQoL. Studies conducted in other European countries have also confirmed similar results, with poorer self-assessments of physical and mental health in patients with LBP [16,54,55,56].

Furthermore, the results of the CORE-OM indicate that in the areas of well-being and problems/symptoms, participants achieve higher values than the bordering ones, which indicates the presence of mental distress. The assumption is that psychical and emotional limitations are unquestionably reflected in the mental distress of the participants, which was confirmed in previous research in the world [16,54]. Lower results in the self-assessments of different health aspects of people with CP are in accordance with the results of many other studies, which stress the burden caused by CP in the overall functioning of a person [16,50,54,55]. Furthermore, the observed difficulties in the area of the well-being and problems/symptoms subscales, despite the absence of a high level of mental distress, indicate that the participants experienced dissatisfaction with themselves and difficulties due to their present CP and other physical difficulties and limitations. The results of relevant research suggest that the presence of CP shares the same pathophysiological pathways with mental distress [57,58,59].

As the average estimated pain intensity in our participants was moderate, which certainly means a certain level of continuous stress, it is possible that despite dissatisfaction and physical difficulties, the determined intensity of the moderate pain present has not yet led to damage that would cause mental disorders [60]. Our results can be related to the fact that most participants were retired, which may affect their lower level of mental distress [60,61], but there is also the possibility that the participants were accepting of their own pain in old age [62]. The participants who had high levels of mental distress noted significant emotional limitations, lower energy, poorer psychological health and social functioning, stronger pain, and poorer general health than participants who had low levels in these dimensions on the CORE-OM. These results clearly indicate the importance of mental distress in the participants’ HRQoL. The complex two-way relationship between pain and mental distress such as depression indicates that pain causes depression and that this results in a stronger pain experience and a lower motivation to perform physical activities, which becomes a vicious cycle that is very difficult to break and get out of [16].

Although the participants did not express high levels of mental distress, which indicates the absence of an unpleasant emotional state in which a person finds it difficult to adapt to environmental requirements and show maladaptive forms of experience and behavior, it is noted that the difficulties identified in the well-being and problems/symptoms dimensions concern quality of life, except for physical limitation and functioning. The reason for this result can be explained by the assumption that participants will, over a number of years of living with CP, create their own defense or compensatory mechanisms in order to function more effectively with as few restrictions as possible. These explanations of defense mechanisms as automatic processes that reduce and mitigate the harmful effects of pain by regulating the emotional response of individuals were given by Valliant in describing adaptive mental mechanisms [63]. Similar results were confirmed in patients with fibromyalgia, who developed significantly stronger defense mechanisms compared to healthy individuals [64]. However, an interesting result is that the area of physical limitation and functioning was significantly negatively associated with the estimated pain intensity. Although it was CP was of a moderate intensity, in combination with old age, it significantly affected the functioning and limitations of the individual [56]. Crofford, in his research, cites a downward physical and emotional spiral called “physical and mental deconditioning” because patients with chronic back pain have a reduced ability to engage in various activities such as work, recreational activities, and interactions with family members and friends [65]. Thus, despite our assumption that over time, participants will find their own defense and compensatory mechanisms, in certain areas that affect overall HRQoL, such as emotional limitation and energy, our participants still experienced significant disruptions. These results are consistent with Norwegian research that examined the association between limitations in physical functioning and mental distress and found a significant association between them, as well as a significant association of low energy with HRQoL [66,67]. Furthermore, the results indicate that the presence of negative thinking in participants was related to their quality of life in such a way that higher levels of negative, risky thinking were reflected in lower levels of energy, psychological health, social functioning, and general health, and vice versa. Similar results have been published in other studies that directly link positive and negative thinking to increasing or decreasing pain levels, which are then reflected in other areas of life [68,69]. The recorded results indicate a milder or stronger connection between mental distress and quality of life, i.e., the level of quality of life and the presence of mental distress are inevitably intertwined. Similar results have been reported in other studies which state that mental distress is a major obstacle to effective pain relief [70], which has important effects on life quality [16]. Further, CP is frequently connected to distress [67] and negative outcomes for mental health such as depression and anxiety [4,71].

In our study, we also found a significant negative association between pain intensity and SF-36 pain, and physical functioning and limitation, while no significant association was found with other areas that included HRQoL. That the presence of pain is significantly related to the level of HRQoL has been confirmed in other studies, as well [32,72]. Moreover, previous studies have found that a higher level of pain intensity and/or limitation is related to a lower HRQoL [73,74,75]. However, as mentioned above, it is possible that patients, especially in the presence of, on average, moderate CP, have brought effective compensation methods to a level that minimally impairs their mental distress and HRQoL [63,64].

There was no significant correlation found between the CORE-OM and pain intensity. The observed results can also be explained by the described defense and/or compensatory mechanisms due to long-term living with pain, as stated by Valliant and Romeo et al., in their research [63,64]. That is, the lack of correlation in our participants could be explained with the results of the research conducted by Gerlde et al., which emphasizes the need to assess both pain and mental distress and not to take it for granted that pain involves great mental stress in an individual [76].

## 5. Implications for Practice

The results of this study confirm previous knowledge about the phenomenon of chronic pain as a diagnosis that affects all aspects of a patient’s life. We confirmed that patients with CLBP have impaired health, both physical and mental, which is in accordance with the international literature. Chronic pain and its consequences impact the quality of life of the patient, regardless of the economic development of a specific territory or culture. Furthermore, the results of this study clearly indicate the need to apply the most modern guidelines for patients with CLBP and to introduce a multidisciplinary and multimodal approach to the treatment of CLBP. In this way, patients with CLBP will be provided with continuous maximum support in overcoming chronic pain and prolonging independence in meeting basic human needs and daily functioning, which will affect the overall quality of life of the patient. In conclusion, confirming the findings from the international literature, our study creates the vital foundations necessary to raise awareness in professionals of these needs, as well as of the administrative and logistical requirements to provide patients with adequate treatment of CLBP through a multidisciplinary and multimodal approach.

## 6. Limitations

According to our plans, future research should address the shortcomings of this study, which means conducting cross-sectional studies with, in general, larger samples and with a greater proportion of men. This study included only participants from one clinical hospital center, which can affect the accuracy of the interpretation of the results. Further, there was an absence of data processing with more complex statistical methods, as well as an absence of qualitative analysis that could contribute to the depth of the results. Moreover, several instruments were used in the research, which could have fatigued the participants and made them less interested in participating in the research and in filling out the questionnaire. Lastly, it is important to be aware that the used questionnaires have biases.

## 7. Conclusions

This research confirmed the existence of poorer physical health in comparison to mental health of participants with CLBP, according to the SF-36 Health Status Questionnaire. The participants’ HRQoL varied according to their level of mental distress, according to Clinical Outcome in Routine Evaluation—Outcome Measures questionnaire. The presence of CLBP was associated with the physical functioning and physical limitations of the participants. The study results indicate a connection between the presence of mental distress and almost all aspects of HRQoL in participants with CLBP.

## Figures and Tables

**Table 1:** Sociodemographic characteristics of the participants (*n* = 148).

Variable		N (%)
Gender	Male	29 (20)
	Female	119 (80)
Age	28–39	12 (8)
	40–49	25 (17)
	50–59	42 (28)
	60–69	43 (29)
	70–79	26 (18)
Education degree	Primary school	35 (24)
	High school	90 (61)
	Bachelor	7 (4)
	Master	16 (11)
Working status	Employed, on temporary sick leave	39 (26)
	Employed, on permanent sick leave	17 (12)
	Unemployed	21 (14)
	Retired	71 (48)
Marital status	Married	110 (75)
	Divorced	11 (7)
	Single	9 (6)
	Widowed	18 (12)
In total		148 (100)

**Table 2:** Distribution of scores regarding the scales of the SF-36, CORE-OM, and VAS (*n* = 148).

Variable	Median (25–75%)	Min–Max
Physical health	28.1 (20.6–35.9)	5.–80.0
Mental health	39.4 (29.4–51.4)	9.4–95.0
SF 36 Physical functioning	35.0 (25.0–50.0)	0.0–90.0
SF 36 Physical limitations	0.0 (0.0–0.0)	0.0–100.0
SF 36 Emotional limitations	0.0 (0.0–33.3)	0.0–100.0
SF 36 Energy	35.0 (30.0–50.0)	5.0–90.0
SF 36 Psychological health	56.0 (44.0–68.0)	12.0–100.0
SF 36 Social functioning	50.0 (37.5–62.5)	0.0–100.0
SF 36 Pain	32.5 (22.5–43.8)	0.0–77.5
SF 36 General health	40.0 (25.0–50.0)	10.0–80.0
CORE-OM Total	1.24 (0.86–1.81)	0.21–2.74
CORE-OM Well-being	1.75 (1.06–2.25)	0.0–3.25
CORE-OM Problems/symptoms	1.79 (1.25–2.42)	0.33–3.75
CORE-OM Functioning	1.25 (0.75–1.58)	0.08–2.83
CORE-OM Risky behaviors	0.17 (0–0.33)	0.0–2.17
VAS	6.5 (5.0–8.0)	3.0–10.0

**Table 3:** Differences in the SF-36 subscales considering high (*n* = 65) and low (*n* = 83) levels of mental distress (CORE-OM) (*n* = 148).

Variable	CORE-OM	Sum of Ranks	Mean Rank	*p* *
SF 36 Physical functioning	Low	6600.0	79.51	0.107
	High	4426.0	68.09	
SF 36 Physical limitations	Low	6462.0	77.85	0.282
	High	4564.0	70.21	
SF 36 Emotional limitations	Low	6944.0	83.66	0.003
	High	4082.0	62.80	
SF 36 Energy	Low	7419.0	89.38	<0.001
	High	3312.0	52.57	
SF 36 Psychological health	Low	7397.0	90.20	<0.001
	High	3188.0	50.60	
SF 36 Social functioning	Low	7390.0	89.03	<0.001
	High	3636.0	55.93	
SF 36 Pain	Low	6876.0	82.84	0.007
	High	4150.0	63.84	
SF 36 General health	Low	7228.5	87.09	<0.001
	High	3649.5	57.02	

Low, value below 1.38; high, value above 1.38; * Mann–Whitney U test.

**Table 4:** Correlations between the CORE-OM dimensions, the subscales of the SF-36, and pain intensity (*n* = 148).

Variable	CORE-OM W	CORE-OM P	COREOM F	COREOM R	CORE-OM Total	SF 36 PL	SF 36 PF	SF 36 EL	SF 36 EN	SF 36 PH	SF 36 SF	SF 36 Pain	SF 36 GH	VAS Average
CORE-OM W	1.00	0.77 *	0.79 *	0.51 *	0.86 *	−0.17	−0.12	−0.37 *	−0.55 *	−0.57 *	−0.43 *	−0.34 *	−0.46 *	0.07
CORE-OM P		1.00	0.78 *	0.58 *	0.93 *	−0.13	−0.14	−0.33 *	−0.47 *	−0.50 *	−0.41 *	−0.29 *	−0.47 *	0.08
CORE-OM F			1.00	0.58 *	0.92 *	−0.11	−0.10	−0.36 *	−0.49 *	−0.54 *	−0.44 *	−0.31 *	−0.47 *	0.06
CORE-OM R				1.00	0.68 *	−0.08	−0.05	−0.13	−0.33 *	−0.28 *	−0.34 *	−0.27 *	−0.36 *	0.07
CORE-OM Total					1.00	−0.15	−0.15	−0.37 *	−0.54 *	−0.57 *	−0.47 *	−0.36 *	−0.51 *	0.07
SF 36 PL						1.00	0.30 *	0.42 *	0.26 *	0.13	0.19 *	0.19 *	0.31 *	−0.19 *
SF 36 PF							1.00	0.16 *	0.35 *	0.26 *	0.33 *	0.35 *	0.44 *	−0.28 **
SF 36 EL								1.00	0.34 *	0.41 *	0.30 *	0.20 *	0.32 *	−0.15
SF 36 EN									1.00	0.61 *	0.57 *	0.50 *	0.59 *	−0.15
SF 36 PH										1.00	0.65 *	0.34 *	0.45 *	−0.10
SF 36 SF											1.00	0.45 *	0.40 *	−0.12
SF 36 Pain												1.00	0.48 *	−0.34 **
SF 36 GH													1.00	−0.16
VAS														1.00

W, well-being; P, problems/symptoms; F, functioning; R, risky behavior; SF-PL, physical limitation; PF, physical functioning; EL, emotional limitation; EN, energy; PH, psychological health; SF, social functioning; GH, general health; *p* < 0.05 *; *p* < 0.01 **.

## Data Availability

The data presented in this study are available upon request from the corresponding author.
